# Supplementary material for: Seed bio-priming with beneficial Trichoderma harzianum alleviates cold stress in maize
Source: PeerJ. 2023 Aug 25;11:e15644. doi: 10.7717/peerj.15644 (PMC10461543; doi:10.7717/peerj.15644)
Supplement: Supplemental Information 1 [file peerj-11-15644-s001.docx]

Student Edition of Statistix 10.0 anova-1-10.sx, 8/13/2021, 10:31:48 AM

**Factorial AOV Table for trait1**

| **Source DF SS MS** | **F** | | **P** |
| --- | --- | --- | --- |
| S 1 0.02005 0.02005 | 4.04 | | 0.0531 |
| V 1 0.14421 0.14421 | 29.03 | | 0.0000 |
| P 3 0.55774 0.18591 | 37.42 | | 0.0000 |
| S*V 1 0.00002 0.00002 | 0.00 | | 0.9563 |
| S*P 3 0.01200 0.00400 | 0.80 | | 0.5004 |
| V*P 3 0.00272 0.00091 | 0.18 | | 0.9074 |
| S*V*P 3 0.00382 0.00127  Error 32 0.15898 0.00497  Total 47 0.89955  Grand Mean 0.3298  CV 21.37  **Factorial AOV Table for trait2** | 0.26 | | 0.8562 |
| **Source DF SS MS** | **F** | | **P** |
| S 1 5418.8 5418.75 | 4128.57 | | 0.0000 |
| V 1 1704.1 1704.08 | 1298.35 | | 0.0000 |
| P 3 3535.5 1178.50 | 897.90 | | 0.0000 |
| S*V 1 320.3 320.33 | 244.06 | | 0.0000 |
| S*P 3 288.4 96.14 | 73.25 | | 0.0000 |
| V*P 3 113.4 37.81 | 28.80 | | 0.0000 |
| S*V*P 3 221.5 73.83  Error 32 42.0 1.31  Total 47 11644.0  Grand Mean 79.500  CV 1.44  **Factorial AOV Table for trait3** | 56.25 | | 0.0000 |
|  |  |  |  |
| **Source DF SS MS** | **F** | **P** | |
| S 1 1722.8 1722.81 | 5.77 | 0.0223 | |
| V 1 2850.0 2850.00 | 9.54 | 0.0041 | |
| P 3 740.7 246.90 | 0.83 | 0.4890 | |
| S*V 1 456.3 456.31 | 1.53 | 0.2255 | |
| S*P 3 62.8 20.94 | 0.07 | 0.9755 | |
| V*P 3 46.1 15.36 | 0.05 | 0.9843 | |
| S*V*P 3 33.5 11.16  Error 32 9558.5 298.70  Total 47 15470.7  Grand Mean 85.699  CV 20.17  **Factorial AOV Table for trait4** | 0.04 | 0.9902 | |
| **Source DF SS MS** | **F** | **P** | |
| S 1 2.62 2.62 | 0.04 | 0.8452 | |
| V 1 1155.03 1155.03 | 17.07 | 0.0002 | |
| P 3 2406.85 802.28 | 11.85 | 0.0000 | |
| S*V 1 208.35 208.35 | 3.08 | 0.0889 | |
| S*P 3 87.99 29.33 | 0.43 | 0.7306 | |
| V*P 3 87.93 29.31 | 0.43 | 0.7308 | |
| S*V*P 3 121.18 40.39  Error 32 2165.63 67.68 Total 47 6235.58 | 0.60 | 0.6217 | |

Grand Mean 40.089

CV 20.52

**Factorial AOV Table for trait5**

| **Source DF SS MS** | **F** | **P** |
| --- | --- | --- |
| S 1 39.69 39.69 | 0.97 | 0.3321 |
| V 1 3236.87 3236.87 | 79.09 | 0.0000 |
| P 3 543.84 181.28 | 4.43 | 0.0103 |
| S*V 1 50.37 50.37 | 1.23 | 0.2756 |
| S*P 3 363.14 121.05 | 2.96 | 0.0471 |
| V*P 3 678.66 226.22 | 5.53 | 0.0036 |
| S*V*P 3 197.34 65.78  Error 32 1309.72 40.93  Total 47 6419.65  Grand Mean 30.278  CV 21.13  **Factorial AOV Table for trait6** | 1.61 | 0.2070 |
| **Source DF SS MS** | **F** | **P** |
| S 1 4.934 4.934 | 2.42 | 0.1294 |
| V 1 297.694 297.694 | 146.21 | 0.0000 |
| P 3 261.073 87.024 | 42.74 | 0.0000 |
| S*V 1 15.221 15.221 | 7.48 | 0.0101 |
| S*P 3 12.136 4.045 | 1.99 | 0.1357 |
| V*P 3 79.062 26.354 | 12.94 | 0.0000 |
| S*V*P 3 9.260 3.087  Error 32 65.156 2.036  Total 47 744.537  Grand Mean 6.0621  CV 23.54  **Factorial AOV Table for trait7** | 1.52 | 0.2292 |
| **Source DF SS MS** | **F** | **P** |
| S 1 0.143 0.143 | 0.06 | 0.8069 |
| V 1 190.898 190.898 | 81.34 | 0.0000 |
| P 3 243.167 81.056 | 34.54 | 0.0000 |
| S*V 1 9.915 9.915 | 4.22 | 0.0481 |
| S*P 3 3.884 1.295 | 0.55 | 0.6507 |
| V*P 3 46.413 15.471 | 6.59 | 0.0014 |
| S*V*P 3 2.849 0.950  Error 32 75.101 2.347  Total 47 572.369  Grand Mean 6.9508  CV 22.04  **Factorial AOV Table for trait8** | 0.40 | 0.7506 |
| **Source DF SS MS** | **F** | **P** |
| S 1 20.22 20.216 | 0.36 | 0.5518 |
| V 1 378.10 378.097 | 6.76 | 0.0140 |
| P 3 1010.03 336.676 | 6.02 | 0.0023 |
| S*V 1 0.79 0.791 | 0.01 | 0.9060 |
| S*P 3 19.46 6.487 | 0.12 | 0.9500 |
| V*P 3 83.95 27.982 | 0.50 | 0.6845 |
| S*V*P 3 164.17 54.722 | 0.98 | 0.4148 |

Error 32 1788.55 55.892 Total 47 3465.25

Grand Mean 34.834

CV 21.46

**Factorial AOV Table for trait9**

| **Source DF SS MS F** | **P** |
| --- | --- |
| S 1 451.04 451.043 1.85 | 0.1835 |
| V 1 969.49 969.487 3.97 | 0.0548 |
| P 3 585.15 195.051 0.80 | 0.5035 |
| S*V 1 19.80 19.802 0.08 | 0.7776 |
| S*P 3 64.96 21.655 0.09 | 0.9657 |
| V*P 3 43.18 14.392 0.06 | 0.9809 |
| S*V*P 3 31.50 10.500 0.04  Error 32 7809.82 244.057  Total 47 9974.95  Grand Mean 77.822  CV 20.07  **Factorial AOV Table for trait10** | 0.9879 |
| **Source DF SS MS F** | **P** |
| S 1 1374.29 1374.29 10.87 | 0.0024 |
| V 1 98.23 98.23 0.78 | 0.3846 |
| P 3 1159.62 386.54 3.06 | 0.0423 |
| S*V 1 64.96 64.96 0.51 | 0.4787 |
| S*P 3 175.39 58.46 0.46 | 0.7104 |
| V*P 3 39.86 13.29 0.11 | 0.9565 |
| S*V*P 3 50.94 16.98 0.13  Error 32 4044.88 126.40  Total 47 7008.16  Grand Mean 55.663  CV 20.20 | 0.9389 |
