# Supplementary material for: Seed bio-priming with beneficial Trichoderma harzianum alleviates cold stress in maize
Source: PeerJ. 2023 Aug 25;11:e15644. doi: 10.7717/peerj.15644 (PMC10461543; doi:10.7717/peerj.15644)
Supplement: Supplemental Information 2 [file peerj-11-15644-s002.docx]

Student Edition of Statistix 10.0 anova-1-10.sx, 8/13/2021, 10:34:12 AM

**Factorial AOV Table for trait11**

| **Source DF SS MS F** | **P** |
| --- | --- |
| S 1 11.2759 11.2759 8.72 | 0.0058 |
| V 1 1.3897 1.3897 1.08 | 0.3076 |
| P 3 33.3768 11.1256 8.61 | 0.0002 |
| S*V 1 0.3039 0.3039 0.24 | 0.6311 |
| S*P 3 1.3434 0.4478 0.35 | 0.7919 |
| V*P 3 1.0558 0.3519 0.27 | 0.8449 |
| S*V*P 3 4.0982 1.3661 1.06  Error 32 41.3652 1.2927  Total 47 94.2088  Grand Mean 5.5871  CV 20.35  **Factorial AOV Table for trait12** | 0.3811 |
| **Source DF SS MS F** | **P** |
| S 1 0.179 0.1788 0.01 | 0.9134 |
| V 1 68.104 68.1038 4.58 | 0.0401 |
| P 3 69.452 23.1507 1.56 | 0.2190 |
| S*V 1 25.693 25.6930 1.73 | 0.1980 |
| S*P 3 2.016 0.6721 0.05 | 0.9870 |
| V*P 3 0.998 0.3327 0.02 | 0.9954 |
| S*V*P 3 2.428 0.8094 0.05  Error 32 475.879 14.8712  Total 47 644.750  Grand Mean 19.190  CV 20.10  **Factorial AOV Table for trait13** | 0.9830 |
| **Source DF SS MS F** | **P** |
| S 1 1070.7 1070.69 18.70 | 0.0001 |
| V 1 2965.7 2965.74 51.80 | 0.0000 |
| P 3 8929.9 2976.63 51.99 | 0.0000 |
| S*V 1 225.8 225.77 3.94 | 0.0557 |
| S*P 3 354.2 118.06 2.06 | 0.1249 |
| V*P 3 977.4 325.81 5.69 | 0.0031 |
| S*V*P 3 364.8 121.59 2.12  Error 32 1832.2 57.26  Total 47 16720.7  Grand Mean 33.394  CV 22.66  **Factorial AOV Table for trait14** | 0.1167 |
| **Source DF SS MS F** | **P** |
| S 1 0.02556 0.02556 5.09 | 0.0310 |
| V 1 0.04620 0.04620 9.21 | 0.0048 |
| P 3 0.40968 0.13656 27.21 | 0.0000 |
| S*V 1 0.00034 0.00034 0.07 | 0.7972 |
| S*P 3 0.01444 0.00481 0.96 | 0.4238 |
| V*P 3 0.05906 0.01969 3.92 | 0.0171 |
| S*V*P 3 0.00028 0.00009 0.02  Error 32 0.16057 0.00502 Total 47 0.71613 | 0.9965 |

Grand Mean 0.3375

CV 20.99

**Factorial AOV Table for Chla**

| **Source DF SS MS** | **F** | **P** |
| --- | --- | --- |
| S 1 78.07 78.073 | 4.04 | 0.0530 |
| V 1 645.49 645.489 | 33.39 | 0.0000 |
| P 3 155.67 51.890 | 2.68 | 0.0632 |
| S*V 1 98.73 98.734 | 5.11 | 0.0308 |
| S*P 3 20.57 6.858 | 0.35 | 0.7860 |
| V*P 3 7.95 2.649 | 0.14 | 0.9372 |
| S*V*P 3 13.91 4.635  Error 32 618.66 19.333  Total 47 1639.05  Grand Mean 21.496  CV 20.45  **Factorial AOV Table for Chlb** | 0.24 | 0.8680 |
| **Source DF SS MS** | **F** | **P** |
| S 1 5.6708 5.6708 | 6.66 | 0.0146 |
| V 1 11.1814 11.1814 | 13.14 | 0.0010 |
| P 3 13.9485 4.6495 | 5.46 | 0.0038 |
| S*V 1 2.6563 2.6563 | 3.12 | 0.0868 |
| S*P 3 0.2984 0.0995 | 0.12 | 0.9496 |
| V*P 3 0.5354 0.1785 | 0.21 | 0.8890 |
| S*V*P 3 4.6539 1.5513  Error 32 27.2361 0.8511  Total 47 66.1807  Grand Mean 4.5240  CV 20.39 | 1.82 | 0.1629 |
| **Factorial AOV Table for carotenoe** | |  |
| **Source DF SS MS F** | | **P** |
| S 1 27 27 0.04 | | 0.8366 |
| V 1 121035 121035 197.28 | | 0.0000 |
| P 3 30852 10284 16.76 | | 0.0000 |
| S*V 1 130 130 0.21 | | 0.6485 |
| S*P 3 2135 712 1.16 | | 0.3401 |
| V*P 3 2348 783 1.28 | | 0.2994 |
| S*V*P 3 861 287 0.47  Error 32 19633 614  Total 47 177021  Grand Mean 109.81  CV 22.56  **Factorial AOV Table for fvm** | | 0.7067 |
| **Source DF SS MS F** | | **P** |
| S 1 0.01044 0.01044 0.42 | | 0.5225 |
| V 1 0.02623 0.02623 1.05 | | 0.3131 |
| P 3 0.03075 0.01025 0.41 | | 0.7466 |
| S*V 1 0.14719 0.14719 5.89 | | 0.0210 |
| S*P 3 0.00052 0.00017 0.01 | | 0.9992 |
| V*P 3 0.00379 0.00126 0.05 | | 0.9847 |
| S*V*P 3 0.00069 0.00023 0.01 | | 0.9988 |

Error 32 0.79915 0.02497 Total 47 1.01876

Grand Mean 0.7873

CV 20.07

**Factorial AOV Table for fo**

| **Source DF SS MS** | **F P** | |
| --- | --- | --- |
| S 1 3888 3888.0 | 3.99 0.0544 | |
| V 1 140 140.1 | 0.14 0.7071 | |
| P 3 84098 28032.7 | 28.76 0.0000 | |
| S*V 1 1452 1452.0 | 1.49 0.2312 | |
| S*P 3 3623 1207.8 | 1.24 0.3117 | |
| V*P 3 4510 1503.4 | 1.54 0.2225 | |
| S*V*P 3 1171 390.4  Error 32 31191 974.7  Total 47 130074  Grand Mean 149.04  CV 20.95  **Factorial AOV Table for fm** | 0.40 0.7535 | |
| **Source DF SS MS** | **F** | **P** |
| S 1 50830 50830 | 2620.67 | 0.0000 |
| V 1 239419 239419 | 12343.82 | 0.0000 |
| P 3 1011257 337086 | 17379.29 | 0.0000 |
| S*V 1 419628 419628 | 21634.96 | 0.0000 |
| S*P 3 197146 65715 | 3388.12 | 0.0000 |
| V*P 3 621314 207105 | 10677.78 | 0.0000 |
| S*V*P 3 646202 215401  Error 32 621 19  Total 47 3186417  Grand Mean 679.83  CV 0.65 | 11105.51 | 0.0000 |
