# Supplementary material for: Seed bio-priming with beneficial Trichoderma harzianum alleviates cold stress in maize
Source: PeerJ. 2023 Aug 25;11:e15644. doi: 10.7717/peerj.15644 (PMC10461543; doi:10.7717/peerj.15644)
Supplement: Supplemental Information 3 [file peerj-11-15644-s003.docx]

Student Edition of Statistix 10.0 8/13/2021, 3:54:19 PM

**LSD All-Pairwise Comparisons Test of trait1 for S**

**S Mean Homogeneous Groups**

control 0.3503 A Cool 0.3094 A

Alpha 0.05 Standard Error for Comparison 0.0203 Critical T Value 2.037 Critical Value for Comparison 0.0414 There are no significant pairwise differences among the means.

**LSD All-Pairwise Comparisons Test of trait1 for V**

**V Mean Homogeneous Groups**

A 0.3846 A k 0.2750 B

Alpha 0.05 Standard Error for Comparison 0.0203 Critical T Value 2.037 Critical Value for Comparison 0.0414 All 2 means are significantly different from one another.

**LSD All-Pairwise Comparisons Test of trait1 for P**

**P Mean Homogeneous Groups**

3 0.4765 A

1. 0.3705 B
2. 0.2897 C

0 0.1825 D

Alpha 0.05 Standard Error for Comparison 0.0288 Critical T Value 2.037 Critical Value for Comparison 0.0586 All 4 means are significantly different from one another.

**LSD All-Pairwise Comparisons Test of trait1 for S*V**

**S V Mean Homogeneous Groups**

control A 0.4045 A Cool A 0.3648 A control k 0.2960 B Cool k 0.2540 B

Alpha 0.05 Standard Error for Comparison 0.0288

Critical T Value 2.037 Critical Value for Comparison 0.0586 There are 2 groups (A and B) in which the means are not significantly different from one another.

**LSD All-Pairwise Comparisons Test of trait1 for S*P**

**S P Mean Homogeneous Groups**

control 3 0.5010 A Cool 3 0.4520 AB Cool 1 0.3765 BC control 1 0.3645 C control 2 0.3255 CD Cool 2 0.2540 DE control 0 0.2100 EF Cool 0 0.1550 F

Alpha 0.05 Standard Error for Comparison 0.0407

Critical T Value 2.037 Critical Value for Comparison 0.0829

There are 6 groups (A, B, etc.) in which the means

are not significantly different from one another.

**LSD All-Pairwise Comparisons Test of trait1 for V*P**

**V P Mean Homogeneous Groups**

A 3 0.5400 A A 1 0.4315 B k 3 0.4130 BC A 2 0.3360 CD k 1 0.3095 DE k 2 0.2435 E A 0 0.2310 E k 0 0.1340 F

Alpha 0.05 Standard Error for Comparison 0.0407

Critical T Value 2.037 Critical Value for Comparison 0.0829 There are 6 groups (A, B, etc.) in which the means are not significantly different from one another.

**LSD All-Pairwise Comparisons Test of trait1 for S*V*P**

**S V P Mean Homogeneous Groups**

control A 3 0.5660 A Cool A 3 0.5140 AB Cool A 1 0.4510 ABC control k 3 0.4360 BC control A 1 0.4120 BCD Cool k 3 0.3900 CDE control A 2 0.3700 CDEF control k 1 0.3170 DEFG Cool A 2 0.3020 DEFGH Cool k 1 0.3020 DEFGH control k 2 0.2810 EFGH control A 0 0.2700 FGH Cool k 2 0.2060 GHI Cool A 0 0.1920 HI control k 0 0.1500 I Cool k 0 0.1180 I

Alpha 0.05 Standard Error for Comparison 0.0576

Critical T Value 2.037 Critical Value for Comparison 0.1172 There are 9 groups (A, B, etc.) in which the means are not significantly different from one another. **LSD All-Pairwise Comparisons Test of trait2 for S**

**S Mean Homogeneous Groups**

control 90.125 A Cool 68.875 B

Alpha 0.05 Standard Error for Comparison 0.3307 Critical T Value 2.037 Critical Value for Comparison 0.6737 All 2 means are significantly different from one another.

**LSD All-Pairwise Comparisons Test of trait2 for V**

**V Mean Homogeneous Groups**

A 85.458 A k 73.542 B

Alpha 0.05 Standard Error for Comparison 0.3307 Critical T Value 2.037 Critical Value for Comparison 0.6737 All 2 means are significantly different from one another.

**LSD All-Pairwise Comparisons Test of trait2 for P**

**P Mean Homogeneous Groups**

3 90.167 A

1. 85.417 B
2. 73.250 C

0 69.167 D

Alpha 0.05 Standard Error for Comparison 0.4677 Critical T Value 2.037 Critical Value for Comparison 0.9527 All 4 means are significantly different from one another.

**LSD All-Pairwise Comparisons Test of trait2 for S*V**

**S V Mean Homogeneous Groups**

control A 93.500 A control k 86.750 B Cool A 77.417 C

Cool k 60.333 D

Alpha 0.05 Standard Error for Comparison 0.4677 Critical T Value 2.037 Critical Value for Comparison 0.9527 All 4 means are significantly different from one another.

**LSD All-Pairwise Comparisons Test of trait2 for S*P**

**S P Mean Homogeneous Groups**

control 3 99.500 A control 1 93.333 B control 2 84.000 C control 0 83.667 C Cool 3 80.833 D

Cool 1 77.500 E

Cool 2 62.500 F

Cool 0 54.667 G

Alpha 0.05 Standard Error for Comparison 0.6614

Critical T Value 2.037 Critical Value for Comparison 1.3473 There are 7 groups (A, B, etc.) in which the means are not significantly different from one another.

**LSD All-Pairwise Comparisons Test of trait2 for V*P**

**V P Mean Homogeneous Groups**

A 3 95.333 A A 1 93.000 B k 3 85.000 C A 2 80.500 D k 1 77.833 E A 0 73.000 F k 2 66.000 G k 0 65.333 G

Alpha 0.05 Standard Error for Comparison 0.6614

Critical T Value 2.037 Critical Value for Comparison 1.3473 There are 7 groups (A, B, etc.) in which the means are not significantly different from one another.

**LSD All-Pairwise Comparisons Test of trait2 for S*V*P**

**S V P Mean Homogeneous Groups**

control k 3 99.667 A control A 3 99.333 A control A 1 97.000 B control A 2 91.333 C Cool A 3 91.333 C control k 1 89.667 CD Cool A 1 89.000 D control A 0 86.333 E control k 0 81.000 F control k 2 76.667 G Cool k 3 70.333 H

Cool A 2 69.667 H

Cool k 1 66.000 I

Cool A 0 59.667 J

Cool k 2 55.333 K

Cool k 0 49.667 L

Alpha 0.05 Standard Error for Comparison 0.9354

Critical T Value 2.037 Critical Value for Comparison 1.9054 There are 12 groups (A, B, etc.) in which the means are not significantly different from one another. **LSD All-Pairwise Comparisons Test of trait3 for S**

**S Mean Homogeneous Groups** Cool 91.690 A control 79.708 B

Alpha 0.05 Standard Error for Comparison 4.9892 Critical T Value 2.037 Critical Value for Comparison 10.163 All 2 means are significantly different from one another.

**LSD All-Pairwise Comparisons Test of trait3 for V**

**V Mean Homogeneous Groups** k 93.405 A A 77.994 B

Alpha 0.05 Standard Error for Comparison 4.9892 Critical T Value 2.037 Critical Value for Comparison 10.163 All 2 means are significantly different from one another.

**LSD All-Pairwise Comparisons Test of trait3 for P**

**P Mean Homogeneous Groups**

1 91.081 A

3 87.804 A

2 82.351 A

0 81.561 A

Alpha 0.05 Standard Error for Comparison 7.0558 Critical T Value 2.037 Critical Value for Comparison 14.372 There are no significant pairwise differences among the means.

**LSD All-Pairwise Comparisons Test of trait3 for S*V**

| **S V** | **Mean** | **Homogeneous Groups** |  |  |
| --- | --- | --- | --- | --- |
| Cool k | 102.48 | A |  |  |
| control k | 84.33 | B |  |  |
| Cool A | 80.90 | B |  |  |
| control A | 75.09 | B |  |  |
| Alpha 0.05 Standard Error for Comparison | | | | 7.0558 |
| Critical T Value 2.037 Critical Value for Comparison There are 2 groups (A and B) in which the means are not significantly different from one another.  **LSD All-Pairwise Comparisons Test of trait3 for S*P**  **S P Mean Homogeneous Groups**  Cool 1 98.497 A  Cool 3 94.350 AB  Cool 0 87.245 AB Cool 2 86.669 AB control 1 83.664 AB control 3 81.259 AB control 2 78.034 B control 0 75.876 B | | | | 14.372 |
| Alpha 0.05 Standard Error for Comparison | | | | 9.9783 |
| Critical T Value 2.037 Critical Value for Comparison There are 2 groups (A and B) in which the means are not significantly different from one another.  **LSD All-Pairwise Comparisons Test of trait3 for V*P**  **V P Mean Homogeneous Groups**  k 1 99.400 A k 3 94.366 AB k 0 90.535 ABC k 2 89.319 ABC A 1 82.761 ABC  A 3 81.243 ABC  A 2 75.384 BC  A 0 72.587 C | | | | 20.325 |
| Alpha 0.05 Standard Error for Comparison | | | | 9.9783 |
| Critical T Value 2.037 Critical Value for Comparison There are 3 groups (A, B, etc.) in which the means are not significantly different from one another.  **LSD All-Pairwise Comparisons Test of trait3 for S*V*P**  **S V P Mean Homogeneous Groups**  Cool k 1 110.28 A  Cool k 3 102.72 AB  Cool k 0 100.31 AB Cool k 2 96.61 ABC control k 1 88.52 ABC Cool A 1 86.72 ABC control k 3 86.01 ABC Cool A 3 85.98 ABC control k 2 82.02 ABC control k 0 80.76 BC control A 1 78.81 BC Cool A 2 76.72 BC control A 3 76.50 BC Cool A 0 74.18 BC control A 2 74.04 BC control A 0 70.99 C | | | | 20.325 |
| Alpha 0.05 Standard Error for Comparison | | | | 14.112 |
| Critical T Value 2.037 Critical Value for Comparison | | | | 28.744 |

There are 3 groups (A, B, etc.) in which the means are not significantly different from one another. **LSD All-Pairwise Comparisons Test of trait4 for S**

**S Mean Homogeneous Groups** Cool 40.322 A control 39.855 A

Alpha 0.05 Standard Error for Comparison 2.3748 Critical T Value 2.037 Critical Value for Comparison 4.8373 There are no significant pairwise differences among the means.

**LSD All-Pairwise Comparisons Test of trait4 for V**

**V Mean Homogeneous Groups** k 44.994 A A 35.183 B

Alpha 0.05 Standard Error for Comparison 2.3748 Critical T Value 2.037 Critical Value for Comparison 4.8373 All 2 means are significantly different from one another.

**LSD All-Pairwise Comparisons Test of trait4 for P**

**P Mean Homogeneous Groups**

1 50.327 A

3 42.258 B

2 36.536 BC

0 31.234 C

Alpha 0.05 Standard Error for Comparison 3.3585

Critical T Value 2.037 Critical Value for Comparison 6.8410 There are 3 groups (A, B, etc.) in which the means are not significantly different from one another.

**LSD All-Pairwise Comparisons Test of trait4 for S*V**

**S V Mean Homogeneous Groups**

control k 46.844 A Cool k 43.144 AB Cool A 37.500 BC control A 32.866 C

Alpha 0.05 Standard Error for Comparison 3.3585

Critical T Value 2.037 Critical Value for Comparison 6.8410 There are 3 groups (A, B, etc.) in which the means are not significantly different from one another.

**LSD All-Pairwise Comparisons Test of trait4 for S*P**

**S P Mean Homogeneous Groups**

control 1 51.926 A Cool 1 48.728 AB control 3 42.346 ABC Cool 3 42.171 BC Cool 2 38.727 CD control 2 34.344 CD Cool 0 31.663 D control 0 30.804 D

Alpha 0.05 Standard Error for Comparison 4.7496 Critical T Value 2.037 Critical Value for Comparison 9.6746 There are 4 groups (A, B, etc.) in which the means are not significantly different from one another.

**LSD All-Pairwise Comparisons Test of trait4 for V*P**

**V P Mean Homogeneous Groups**

k 1 55.018 A k 3 46.990 AB A 1 45.636 AB k 2 43.529 BC A 3 37.527 BCD k 0 34.439 CD A 2 29.542 D

A 0 28.028 D

Alpha 0.05 Standard Error for Comparison 4.7496

Critical T Value 2.037 Critical Value for Comparison 9.6746 There are 4 groups (A, B, etc.) in which the means are not significantly different from one another.

**LSD All-Pairwise Comparisons Test of trait4 for S*V*P**

**S V P Mean Homogeneous Groups**

control k 1 60.615 A Cool k 1 49.421 AB

Cool A 1 48.035 AB Cool k 3 47.295 ABC control k 3 46.685 BC Cool k 2 43.627 BCD control k 2 43.432 BCD control A 1 43.237 BCD control A 3 38.007 BCDE Cool A 3 37.047 BCDE control k 0 36.644 BCDE Cool A 2 33.827 CDE

Cool k 0 32.235 DE Cool A 0 31.092 DE control A 2 25.257 E control A 0 24.964 E

Alpha 0.05 Standard Error for Comparison 6.7169

Critical T Value 2.037 Critical Value for Comparison 13.682 There are 5 groups (A, B, etc.) in which the means are not significantly different from one another. **LSD All-Pairwise Comparisons Test of trait5 for S**

**S Mean Homogeneous Groups** Cool 31.188 A control 29.369 A

Alpha 0.05 Standard Error for Comparison 1.8468 Critical T Value 2.037 Critical Value for Comparison 3.7618 There are no significant pairwise differences among the means.

**LSD All-Pairwise Comparisons Test of trait5 for V**

**V Mean Homogeneous Groups**

A 38.490 A k 22.066 B Alpha 0.05 Standard Error for Comparison 1.8468 Critical T Value 2.037 Critical Value for Comparison 3.7618 All 2 means are significantly different from one another.

**LSD All-Pairwise Comparisons Test of trait5 for P**

**P Mean Homogeneous Groups**

3 34.925 A

1. 31.382 AB
2. 29.130 BC

0 25.675 C

Alpha 0.05 Standard Error for Comparison 2.6118

Critical T Value 2.037 Critical Value for Comparison 5.3201 There are 3 groups (A, B, etc.) in which the means are not significantly different from one another.

**LSD All-Pairwise Comparisons Test of trait5 for S*V**

**S V Mean Homogeneous Groups**

control A 38.605 A Cool A 38.375 A Cool k 24.000 B control k 20.132 B

Alpha 0.05 Standard Error for Comparison 2.6118

Critical T Value 2.037 Critical Value for Comparison 5.3201 There are 2 groups (A and B) in which the means are not significantly different from one another.

**LSD All-Pairwise Comparisons Test of trait5 for S*P**

**S P Mean Homogeneous Groups**

control 3 37.050 A Cool 3 32.800 AB

Cool 1 32.150 AB Cool 0 31.000 AB control 1 30.615 AB control 2 29.460 B Cool 2 28.800 B control 0 20.350 C

Alpha 0.05 Standard Error for Comparison 3.6936

Critical T Value 2.037 Critical Value for Comparison 7.5237 There are 3 groups (A, B, etc.) in which the means are not significantly different from one another.

**LSD All-Pairwise Comparisons Test of trait5 for V*P**

**V P Mean Homogeneous Groups**

A 3 45.750 A

A 1 41.800 A

A 2 39.010 A A 0 27.400 B k 3 24.100 BC k 0 23.950 BC k 1 20.965 BC k 2 19.250 C

Alpha 0.05 Standard Error for Comparison 3.6936

Critical T Value 2.037 Critical Value for Comparison 7.5237

There are 3 groups (A, B, etc.) in which the means

are not significantly different from one another.

**LSD All-Pairwise Comparisons Test of trait5 for S*V*P**

| **S V P** | **Mean** | **Homogeneous Groups** |  |
| --- | --- | --- | --- |
| Cool A 3 | 45.800 | A |  |
| control A 3 | 45.700 | A |  |
| control A 1 | 42.000 | AB |  |
| Cool A 1 | 41.600 | AB |  |
| control A 2 | 41.320 | AB |  |
| Cool A 2 | 36.700 | ABC |  |
| Cool k 0 | 32.600 | BCD |  |
| Cool A 0 | 29.400 | CDE |  |
| control k 3 | 28.400 | CDE |  |
| control A 0 | 25.400 | DEF |  |
| Cool k 1 | 22.700 | DEF |  |
| Cool k 2 | 20.900 | EF |  |
| Cool k 3 | 19.800 | EF |  |
| control k 1 | 19.230 | EF |  |
| control k 2 | 17.600 | F |  |
| control k 0 | 15.300 | F |  |
| Alpha | 0.05 Standard Error for Comparison | | 5.2236 |

Critical T Value 2.037 Critical Value for Comparison 10.640 There are 6 groups (A, B, etc.) in which the means are not significantly different from one another. **LSD All-Pairwise Comparisons Test of trait6 for S**

**S Mean Homogeneous Groups** Cool 6.3827 A control 5.7415 A

Alpha 0.05 Standard Error for Comparison 0.4119 Critical T Value 2.037 Critical Value for Comparison 0.8391 There are no significant pairwise differences among the means.

**LSD All-Pairwise Comparisons Test of trait6 for V**

**V Mean Homogeneous Groups**

A 8.5525 A k 3.5718 B

Alpha 0.05 Standard Error for Comparison 0.4119 Critical T Value 2.037 Critical Value for Comparison 0.8391 All 2 means are significantly different from one another.

**LSD All-Pairwise Comparisons Test of trait6 for P**

**P Mean Homogeneous Groups**

3 9.1400 A

1. 6.7405 B
2. 5.7280 B

0 2.6400 C

Alpha 0.05 Standard Error for Comparison 0.5825

Critical T Value 2.037 Critical Value for Comparison 1.1866 There are 3 groups (A, B, etc.) in which the means are not significantly different from one another.

**LSD All-Pairwise Comparisons Test of trait6 for S*V**

| **S V Mean Homogeneous Groups**  control A 8.7950 A Cool A 8.3100 A Cool k 4.4555 B control k 2.6880 C |  |
| --- | --- |
| Alpha 0.05 Standard Error for Comparison | 0.5825 |
| Critical T Value 2.037 Critical Value for Comparison There are 3 groups (A, B, etc.) in which the means are not significantly different from one another.  **LSD All-Pairwise Comparisons Test of trait6 for S*P**  **S P Mean Homogeneous Groups** Cool 3 10.210 A control 3 8.070 B control 1 7.050 BC Cool 1 6.431 BCD Cool 2 6.140 CD control 2 5.316 D Cool 0 2.750 E control 0 2.530 E | 1.1866 |
| Alpha 0.05 Standard Error for Comparison | 0.8238 |
| Critical T Value 2.037 Critical Value for Comparison There are 5 groups (A, B, etc.) in which the means are not significantly different from one another.  **LSD All-Pairwise Comparisons Test of trait6 for V*P**  **V P Mean Homogeneous Groups**  A 3 12.380 A  A 1 10.550 B A 2 8.220 C k 3 5.900 D k 2 3.236 E A 0 3.060 E k 1 2.931 E k 0 2.220 E | 1.6781 |
| Alpha 0.05 Standard Error for Comparison | 0.8238 |
| Critical T Value 2.037 Critical Value for Comparison There are 5 groups (A, B, etc.) in which the means are not significantly different from one another.  **LSD All-Pairwise Comparisons Test of trait6 for S*V*P**  **S V P Mean Homogeneous Groups**  control A 3 12.560 A Cool A 3 12.200 A control A 1 11.480 AB Cool A 1 9.620 BC  Cool A 2 8.540 C Cool k 3 8.220 C control A 2 7.900 C Cool k 2 3.740 D control k 3 3.580 D Cool k 1 3.242 D control A 0 3.240 D Cool A 0 2.880 D control k 2 2.732 D control k 1 2.620 D | 1.6781 |

Cool k 0 2.620 D control k 0 1.820 D

Alpha 0.05 Standard Error for Comparison 1.1651

Critical T Value 2.037 Critical Value for Comparison 2.3732 There are 4 groups (A, B, etc.) in which the means are not significantly different from one another. **LSD All-Pairwise Comparisons Test of trait7 for S**

**S Mean Homogeneous Groups** Cool 7.0053 A control 6.8962 A

Alpha 0.05 Standard Error for Comparison 0.4422 Critical T Value 2.037 Critical Value for Comparison 0.9008 There are no significant pairwise differences among the means.

**LSD All-Pairwise Comparisons Test of trait7 for V**

**V Mean Homogeneous Groups**

A 8.9450 A k 4.9565 B

Alpha 0.05 Standard Error for Comparison 0.4422 Critical T Value 2.037 Critical Value for Comparison 0.9008 All 2 means are significantly different from one another.

**LSD All-Pairwise Comparisons Test of trait7 for P**

**P Mean Homogeneous Groups**

3 9.9080 A

1. 7.6250 B
2. 6.6300 B

0 3.6400 C

Alpha 0.05 Standard Error for Comparison 0.6254

Critical T Value 2.037 Critical Value for Comparison 1.2739 There are 3 groups (A, B, etc.) in which the means are not significantly different from one another.

**LSD All-Pairwise Comparisons Test of trait7 for S*V**

**S V Mean Homogeneous Groups**

control A 9.3450 A Cool A 8.5450 A Cool k 5.4655 B control k 4.4475 B

Alpha 0.05 Standard Error for Comparison 0.6254

Critical T Value 2.037 Critical Value for Comparison 1.2739 There are 2 groups (A and B) in which the means are not significantly different from one another.

**LSD All-Pairwise Comparisons Test of trait7 for S*P**

**S P Mean Homogeneous Groups**

control 3 10.210 A Cool 3 9.606 AB Cool 1 8.070 BC control 1 7.180 C Cool 2 6.815 C control 2 6.445 C control 0 3.750 D Cool 0 3.530 D

Alpha 0.05 Standard Error for Comparison 0.8845

Critical T Value 2.037 Critical Value for Comparison 1.8016 There are 4 groups (A, B, etc.) in which the means are not significantly different from one another.

**LSD All-Pairwise Comparisons Test of trait7 for V*P**

**V P Mean Homogeneous Groups**

A 3 11.840 A

A 1 10.590 AB A 2 9.290 BC k 3 7.976 C k 1 4.660 D A 0 4.060 D k 2 3.970 D k 0 3.220 D

Alpha 0.05 Standard Error for Comparison 0.8845

Critical T Value 2.037 Critical Value for Comparison 1.8016 There are 4 groups (A, B, etc.) in which the means are not significantly different from one another.

**LSD All-Pairwise Comparisons Test of trait7 for S*V*P**

**S V P Mean Homogeneous Groups**

control A 3 12.200 A Cool A 3 11.480 A control A 1 10.620 AB Cool A 1 10.560 AB control A 2 9.680 ABC Cool A 2 8.900 BC control k 3 8.220 BC Cool k 3 7.732 CD Cool k 1 5.580 DE control A 0 4.880 EF Cool k 2 4.730 EF Cool k 0 3.820 EF control k 1 3.740 EF Cool A 0 3.240 EF control k 2 3.210 EF control k 0 2.620 F

Alpha 0.05 Standard Error for Comparison 1.2508

Critical T Value 2.037 Critical Value for Comparison 2.5479 There are 6 groups (A, B, etc.) in which the means are not significantly different from one another. **LSD All-Pairwise Comparisons Test of trait8 for S**

**S Mean Homogeneous Groups**

control 35.483 A Cool 34.185 A

Alpha 0.05 Standard Error for Comparison 2.1582 Critical T Value 2.037 Critical Value for Comparison 4.3960 There are no significant pairwise differences among the means.

**LSD All-Pairwise Comparisons Test of trait8 for V**

**V Mean Homogeneous Groups** k 37.641 A A 32.028 B

Alpha 0.05 Standard Error for Comparison 2.1582 Critical T Value 2.037 Critical Value for Comparison 4.3960 All 2 means are significantly different from one another.

**LSD All-Pairwise Comparisons Test of trait8 for P**

**P Mean Homogeneous Groups**

1 41.064 A

3 35.215 A

2 34.946 A

0 28.111 B

Alpha 0.05 Standard Error for Comparison 3.0521

Critical T Value 2.037 Critical Value for Comparison 6.2169 There are 2 groups (A and B) in which the means are not significantly different from one another.

**LSD All-Pairwise Comparisons Test of trait8 for S*V**

**S V Mean Homogeneous Groups**

control k 38.161 A Cool k 37.120 AB control A 32.805 AB Cool A 31.250 B

Alpha 0.05 Standard Error for Comparison 3.0521

Critical T Value 2.037 Critical Value for Comparison 6.2169 There are 2 groups (A and B) in which the means are not significantly different from one another.

**LSD All-Pairwise Comparisons Test of trait8 for S*P**

**S P Mean Homogeneous Groups**

control 1 41.522 A Cool 1 40.607 A control 3 35.288 AB control 2 35.287 AB Cool 3 35.143 ABC Cool 2 34.606 ABC control 0 29.836 BC Cool 0 26.386 C

Alpha 0.05 Standard Error for Comparison 4.3163

Critical T Value 2.037 Critical Value for Comparison 8.7921 There are 3 groups (A, B, etc.) in which the means are not significantly different from one another.

**LSD All-Pairwise Comparisons Test of trait8 for V*P**

**V P Mean Homogeneous Groups**

k 1 44.098 A k 3 39.158 AB k 2 38.608 AB A 1 38.030 AB

A 2 31.285 BC A 3 31.273 BC k 0 28.699 C A 0 27.523 C

| Alpha 0.05 Standard Error for Comparison | | | 4.3163 |
| --- | --- | --- | --- |
| Critical T Value 2.037 Critical Value for Comparison There are 3 groups (A, B, etc.) in which the means are not significantly different from one another.  **LSD All-Pairwise Comparisons Test of trait8 for S*V*P** | | | 8.7921 |
| **S V P** | **Mean Homogeneous Groups** | |  |
| control k 1 | 47.012 A |  |  |
| Cool k 1 | 41.184 AB |  |  |
| Cool k 2 | 41.023 AB |  |  |
| Cool A 1 | 40.029 ABC |  |  |
| Cool k 3 | 39.412 ABC |  |  |
| control k 3 | 38.904 ABCD |  |  |
| control k 2 | 36.193 ABCDE |  |  |
| control A 1 | 36.031 ABCDE |  |  |
| control A 2 | 34.381 BCDE |  |  |
| control A 3 | 31.672 BCDE |  |  |
| Cool A 3 | 30.873 BCDE |  |  |
| control k 0 | 30.536 BCDE |  |  |
| control A 0 | 29.136 BCDE |  |  |
| Cool A 2 | 28.189 CDE |  |  |
| Cool k 0 | 26.862 DE |  |  |
| Cool A 0 | 25.910 E |  |  |
| Alpha | 0.05 | Standard Error for Comparison | 6.1042 |
| Critical T Value 2.037 | | Critical Value for Comparison | 12.434 |
| There are 5 groups (A, B, etc.) in which the means are not significantly different from one another. **LSD All-Pairwise Comparisons Test of trait9 for S**  **S Mean Homogeneous Groups** Cool 80.888 A control 74.757 A | | |  |
| Alpha 0.05 Standard Error for Comparison | | | 4.5098 |
| Critical T Value 2.037 Critical Value for Comparison | | | 9.1861 |

There are no significant pairwise differences among the means.

**LSD All-Pairwise Comparisons Test of trait9 for V**

**V Mean Homogeneous Groups** k 82.317 A A 73.328 A

Alpha 0.05 Standard Error for Comparison 4.5098 Critical T Value 2.037 Critical Value for Comparison 9.1861 There are no significant pairwise differences among the means.

**LSD All-Pairwise Comparisons Test of trait9 for P**

**P Mean Homogeneous Groups**

1 82.775 A

3 79.420 A

2 74.876 A

0 74.217 A

Alpha 0.05 Standard Error for Comparison 6.3778 Critical T Value 2.037 Critical Value for Comparison 12.991 There are no significant pairwise differences among the means.

**LSD All-Pairwise Comparisons Test of trait9 for S*V**

| **S V** | **Mean** | **Homogeneous Groups** |  |
| --- | --- | --- | --- |
| Cool k | 86.024 | A |  |
| control k | 78.609 | AB |  |
| Cool A | 75.751 | AB |  |
| control A | 70.905 | B |  |
| Alpha |  | 0.05 Standard Error for Comparison | 6.3778 |

Critical T Value 2.037 Critical Value for Comparison 12.991 There are 2 groups (A and B) in which the means are not significantly different from one another.

**LSD All-Pairwise Comparisons Test of trait9 for S*P**

| **S P** | **Mean** | **Homogeneous Groups** | |  |
| --- | --- | --- | --- | --- |
| Cool 1 | 87.498 | A |  |  |
| Cool 3 | 82.792 | A |  |  |
| control 1 | 78.053 | A |  |  |
| Cool 0 | 76.871 | A |  |  |
| Cool 2 | 76.391 | A |  |  |
| control 3 | 76.049 | A |  |  |
| control 2 | 73.362 | A |  |  |
| control 0 | 71.564 | A |  |  |
| Alpha |  | 0.05 | Standard Error for Comparison | 9.0196 |

Critical T Value 2.037 Critical Value for Comparison 18.372 There are no significant pairwise differences among the means.

**LSD All-Pairwise Comparisons Test of trait9 for V*P**

**V P Mean Homogeneous Groups**

k 1 88.250 A k 3 82.805 AB k 0 79.612 AB k 2 78.599 AB A 1 77.301 AB

A 3 76.036 AB

A 2 71.153 AB

A 0 68.822 B

Alpha 0.05 Standard Error for Comparison 9.0196

Critical T Value 2.037 Critical Value for Comparison 18.372 There are 2 groups (A and B) in which the means are not significantly different from one another.

**LSD All-Pairwise Comparisons Test of trait9 for S*V*P**

| **S V P** | **Mean** | **Homogeneous Groups** |  |  |
| --- | --- | --- | --- | --- |
| Cool k 1 | 94.398 | A |  |  |
| Cool k 3 | 85.599 | AB |  |  |
| Cool k 0 | 83.589 | AB |  |  |
| control k 1 | 82.101 | AB |  |  |
| Cool A 1 | 80.597 | AB |  |  |
| Cool k 2 | 80.512 | AB |  |  |
| control k 3 | 80.011 | AB |  |  |
| Cool A 3 | 79.985 | AB |  |  |
| control k 2 | 76.687 | AB |  |  |
| control k 0 | 75.636 | AB |  |  |
| control A 1 | 74.005 | AB |  |  |
| Cool A 2 72.270 AB control A 3 72.087 AB Cool A 0 70.154 AB control A 2 70.037 AB control A 0 67.491 B | | | |  |
| Alpha 0.05 Standard Error for Comparison | | | | 12.756 |
| Critical T Value 2.037 Critical Value for Comparison There are 2 groups (A and B) in which the means are not significantly different from one another. **LSD All-Pairwise Comparisons Test of trait10 for S**  **S Mean Homogeneous Groups** Cool 61.013 A control 50.312 B | | | | 25.982 |
| Alpha 0.05 Standard Error for Comparison | | | | 3.2455 |
| Critical T Value 2.037 Critical Value for Comparison All 2 means are significantly different from one another.  **LSD All-Pairwise Comparisons Test of trait10 for V**  **V Mean Homogeneous Groups** k 57.093 A A 54.232 A | | | | 6.6110 |
| Alpha 0.05 Standard Error for Comparison | | | | 3.2455 |
| Critical T Value 2.037 Critical Value for Comparison | | | | 6.6110 |

There are no significant pairwise differences among the means.

**LSD All-Pairwise Comparisons Test of trait10 for P**

**P Mean Homogeneous Groups**

1 61.885 A

3 58.553 A

2 53.113 AB

0 49.100 B

Alpha 0.05 Standard Error for Comparison 4.5899

Critical T Value 2.037 Critical Value for Comparison 9.3493 There are 2 groups (A and B) in which the means are not significantly different from one another.

**LSD All-Pairwise Comparisons Test of trait10 for S*V**

| **S V** | **Mean** | **Homogeneous Groups** |  |
| --- | --- | --- | --- |
| Cool k | 63.607 | A |  |
| Cool A | 58.420 | AB |  |
| control k | 50.579 | B |  |
| control A | 50.045 | B |  |
| Alpha |  | 0.05 Standard Error for Comparison | 4.5899 |

Critical T Value 2.037 Critical Value for Comparison 9.3493 There are 2 groups (A and B) in which the means are not significantly different from one another.

**LSD All-Pairwise Comparisons Test of trait10 for S*P**

**S P Mean Homogeneous Groups**

Cool 1 69.320 A

Cool 3 65.615 AB

| Cool 2 56.302 ABC control 1 54.450 BC Cool 0 52.817 BC control 3 51.491 C control 2 49.923 C control 0 45.384 C |  |
| --- | --- |
| Alpha 0.05 Standard Error for Comparison | 6.4911 |
| Critical T Value 2.037 Critical Value for Comparison There are 3 groups (A, B, etc.) in which the means are not significantly different from one another.  **LSD All-Pairwise Comparisons Test of trait10 for V*P**  **V P Mean Homogeneous Groups**  k 1 63.541 A k 3 61.250 AB A 1 60.229 AB A 3 55.856 AB k 2 54.324 AB A 2 51.901 AB k 0 49.258 B A 0 48.942 B | 13.222 |
| Alpha 0.05 Standard Error for Comparison | 6.4911 |
| Critical T Value 2.037 Critical Value for Comparison There are 2 groups (A and B) in which the means are not significantly different from one another.  **LSD All-Pairwise Comparisons Test of trait10 for S*V*P**  **S V P Mean Homogeneous Groups**  Cool k 1 72.685 A  Cool k 3 69.769 AB  Cool A 1 65.955 ABC  Cool A 3 61.460 ABCD  Cool k 2 56.932 ABCD  Cool A 2 55.672 ABCD Cool k 0 55.043 ABCD control A 1 54.502 ABCD control k 1 54.397 ABCD control k 3 52.730 BCD control k 2 51.715 BCD Cool A 0 50.591 CD control A 3 50.251 CD control A 2 48.131 CD control A 0 47.294 CD control k 0 43.474 D | 13.222 |
| Alpha 0.05 Standard Error for Comparison | 9.1798 |
| Critical T Value 2.037 Critical Value for Comparison | 18.699 |

There are 4 groups (A, B, etc.) in which the means are not significantly different from one another.
