# Supplementary material for: Seed bio-priming with beneficial Trichoderma harzianum alleviates cold stress in maize
Source: PeerJ. 2023 Aug 25;11:e15644. doi: 10.7717/peerj.15644 (PMC10461543; doi:10.7717/peerj.15644)
Supplement: Supplemental Information 4 [file peerj-11-15644-s004.docx]

Student Edition of Statistix 10.0 8/13/2021, 3:55:00 PM

**LSD All-Pairwise Comparisons Test of trait11 for S**

**S Mean Homogeneous Groups** Cool 6.0718 A control 5.1024 B

Alpha 0.05 Standard Error for Comparison 0.3282 Critical T Value 2.037 Critical Value for Comparison 0.6685 All 2 means are significantly different from one another.

**LSD All-Pairwise Comparisons Test of trait11 for V**

**V Mean Homogeneous Groups** k 5.7572 A A 5.4169 A

Alpha 0.05 Standard Error for Comparison 0.3282 Critical T Value 2.037 Critical Value for Comparison 0.6685 There are no significant pairwise differences among the means.

**LSD All-Pairwise Comparisons Test of trait11 for P**

**P Mean Homogeneous Groups**

1 6.4982 A

3 6.2870 A

2 5.0677 B

0 4.4955 B

Alpha 0.05 Standard Error for Comparison 0.4642

Critical T Value 2.037 Critical Value for Comparison 0.9455 There are 2 groups (A and B) in which the means are not significantly different from one another.

**LSD All-Pairwise Comparisons Test of trait11 for S*V**

**S V Mean Homogeneous Groups**

Cool k 6.1624 A Cool A 5.9812 A control k 5.3521 AB control A 4.8527 B

Alpha 0.05 Standard Error for Comparison 0.4642

Critical T Value 2.037 Critical Value for Comparison 0.9455 There are 2 groups (A and B) in which the means are not significantly different from one another.

**LSD All-Pairwise Comparisons Test of trait11 for S*P**

**S P Mean Homogeneous Groups**

Cool 1 7.2504 A Cool 3 6.6940 AB control 3 5.8801 BC control 1 5.7460 BC Cool 2 5.3671 BC Cool 0 4.9756 CD control 2 4.7682 CD control 0 4.0154 D

Alpha 0.05 Standard Error for Comparison 0.6564 Critical T Value 2.037 Critical Value for Comparison 1.3371 There are 4 groups (A, B, etc.) in which the means are not significantly different from one another.

**LSD All-Pairwise Comparisons Test of trait11 for V*P**

**V P Mean Homogeneous Groups**

k 1 6.6993 A k 3 6.4256 A A 1 6.2971 A A 3 6.1484 A k 2 5.4455 AB A 2 4.6898 B A 0 4.5324 B k 0 4.4586 B

Alpha 0.05 Standard Error for Comparison 0.6564

Critical T Value 2.037 Critical Value for Comparison 1.3371 There are 2 groups (A and B) in which the means are not significantly different from one another.

**LSD All-Pairwise Comparisons Test of trait11 for S*V*P**

**S V P Mean Homogeneous Groups**

Cool k 1 7.4885 A

Cool A 1 7.0123 AB

Cool k 3 6.8777 AB Cool A 3 6.5102 ABC control k 3 5.9736 ABCD Cool k 2 5.9212 ABCD control k 1 5.9101 ABCD control A 3 5.7867 ABCD Cool A 0 5.5891 BCD control A 1 5.5818 BCD control k 2 4.9698 CDE Cool A 2 4.8131 CDE control A 2 4.5665 DE control k 0 4.5550 DE Cool k 0 4.3621 DE control A 0 3.4758 E

Alpha 0.05 Standard Error for Comparison 0.9283

Critical T Value 2.037 Critical Value for Comparison 1.8909 There are 5 groups (A, B, etc.) in which the means are not significantly different from one another. **LSD All-Pairwise Comparisons Test of trait12 for S**

**S Mean Homogeneous Groups**

control 19.251 A Cool 19.129 A

Alpha 0.05 Standard Error for Comparison 1.1132 Critical T Value 2.037 Critical Value for Comparison 2.2676 There are no significant pairwise differences among the means.

**LSD All-Pairwise Comparisons Test of trait12 for V**

**V Mean Homogeneous Groups**

A 20.381 A k 17.999 B

Alpha 0.05 Standard Error for Comparison 1.1132 Critical T Value 2.037 Critical Value for Comparison 2.2676 All 2 means are significantly different from one another.

**LSD All-Pairwise Comparisons Test of trait12 for P**

**P Mean Homogeneous Groups**

1 20.544 A

3 20.057 A

2 18.686 A

0 17.473 A

Alpha 0.05 Standard Error for Comparison 1.5743 Critical T Value 2.037 Critical Value for Comparison 3.2068 There are no significant pairwise differences among the means.

**LSD All-Pairwise Comparisons Test of trait12 for S*V**

| **S V** | **Mean** | **Homogeneous Groups** |  |
| --- | --- | --- | --- |
| Cool A | 21.052 | A |  |
| control A | 19.711 | AB |  |
| control k | 18.792 | AB |  |
| Cool k | 17.206 | B |  |
| Alpha |  | 0.05 Standard Error for Comparison | 1.5743 |

Critical T Value 2.037 Critical Value for Comparison 3.2068 There are 2 groups (A and B) in which the means are not significantly different from one another.

**LSD All-Pairwise Comparisons Test of trait12 for S*P**

| **S P** | **Mean** | **Homogeneous Groups** | |  |
| --- | --- | --- | --- | --- |
| control 1 | 20.881 | A |  |  |
| Cool 3 | 20.213 | A |  |  |
| Cool 1 | 20.208 | A |  |  |
| control 3 | 19.901 | A |  |  |
| control 2 | 18.865 | A |  |  |
| Cool 2 | 18.508 | A |  |  |
| Cool 0 | 17.589 | A |  |  |
| control 0 | 17.357 | A |  |  |
| Alpha |  | 0.05 | Standard Error for Comparison | 2.2264 |

Critical T Value 2.037 Critical Value for Comparison 4.5351 There are no significant pairwise differences among the means.

**LSD All-Pairwise Comparisons Test of trait12 for V*P**

**V P Mean Homogeneous Groups**

A 1 21.589 A

A 3 21.138 A A 2 19.915 AB k 1 19.500 AB k 3 18.976 AB A 0 18.883 AB k 2 17.458 AB k 0 16.063 B

Alpha 0.05 Standard Error for Comparison 2.2264

Critical T Value 2.037 Critical Value for Comparison 4.5351 There are 2 groups (A and B) in which the means are not significantly different from one another.

**LSD All-Pairwise Comparisons Test of trait12 for S*V*P**

**S V P Mean Homogeneous Groups**

Cool A 1 22.167 A Cool A 3 21.977 A control A 1 21.010 AB control k 1 20.752 AB control A 3 20.300 AB Cool A 2 20.120 AB Cool A 0 19.944 AB control A 2 19.710 AB control k 3 19.503 AB Cool k 3 18.449 AB Cool k 1 18.249 AB control k 2 18.020 AB control A 0 17.823 AB Cool k 2 16.895 AB control k 0 16.892 AB Cool k 0 15.233 B

Alpha 0.05 Standard Error for Comparison 3.1487

Critical T Value 2.037 Critical Value for Comparison 6.4136 There are 2 groups (A and B) in which the means are not significantly different from one another. **LSD All-Pairwise Comparisons Test of trait13 for S**

**S Mean Homogeneous Groups**

control 38.117 A Cool 28.671 B

Alpha 0.05 Standard Error for Comparison 2.1844 Critical T Value 2.037 Critical Value for Comparison 4.4494 All 2 means are significantly different from one another.

**LSD All-Pairwise Comparisons Test of trait13 for V**

**V Mean Homogeneous Groups**

A 41.254 A k 25.533 B

Alpha 0.05 Standard Error for Comparison 2.1844 Critical T Value 2.037 Critical Value for Comparison 4.4494 All 2 means are significantly different from one another.

**LSD All-Pairwise Comparisons Test of trait13 for P**

**P Mean Homogeneous Groups**

3 46.700 A

1. 46.400 A
2. 25.325 B

0 15.150 C

Alpha 0.05 Standard Error for Comparison 3.0891

Critical T Value 2.037 Critical Value for Comparison 6.2924 There are 3 groups (A, B, etc.) in which the means are not significantly different from one another.

**LSD All-Pairwise Comparisons Test of trait13 for S*V**

**S V Mean Homogeneous Groups**

control A 43.808 A Cool A 38.700 AB control k 32.425 B Cool k 18.642 C

| Alpha 0.05 Standard Error for Comparison | 3.0891 |
| --- | --- |
| Critical T Value 2.037 Critical Value for Comparison There are 3 groups (A, B, etc.) in which the means are not significantly different from one another.  **LSD All-Pairwise Comparisons Test of trait13 for S*P**  **S P Mean Homogeneous Groups**  control 3 54.567 A control 1 53.150 A Cool 1 39.650 B Cool 3 38.833 B control 2 28.500 C Cool 2 22.150 CD control 0 16.250 D Cool 0 14.050 D | 6.2924 |
| Alpha 0.05 Standard Error for Comparison | 4.3687 |
| Critical T Value 2.037 Critical Value for Comparison There are 4 groups (A, B, etc.) in which the means are not significantly different from one another.  **LSD All-Pairwise Comparisons Test of trait13 for V*P**  **V P Mean Homogeneous Groups**  A 1 59.000 A A 3 58.517 A k 3 34.883 B k 1 33.800 B A 2 30.500 B k 2 20.150 C A 0 17.000 C k 0 13.300 C | 8.8988 |
| Alpha 0.05 Standard Error for Comparison | 4.3687 |
| Critical T Value 2.037 Critical Value for Comparison There are 3 groups (A, B, etc.) in which the means are not significantly different from one another.  **LSD All-Pairwise Comparisons Test of trait13 for S*V*P**  **S V P Mean Homogeneous Groups**  control A 1 61.300 A control A 3 61.133 A Cool A 1 56.700 AB Cool A 3 55.900 AB control k 3 48.000 B control k 1 45.000 BC control A 2 35.000 CD Cool A 2 26.000 DE Cool k 1 22.600 DEF control k 2 22.000 EF Cool k 3 21.767 EF Cool k 2 18.300 EF control A 0 17.800 EF Cool A 0 16.200 EF control k 0 14.700 EF Cool k 0 11.900 F | 8.8988 |

Alpha 0.05 Standard Error for Comparison 6.1783

Critical T Value 2.037 Critical Value for Comparison 12.585 There are 6 groups (A, B, etc.) in which the means are not significantly different from one another. **LSD All-Pairwise Comparisons Test of trait14 for S**

**S Mean Homogeneous Groups**

control 0.3605 A Cool 0.3144 B

Alpha 0.05 Standard Error for Comparison 0.0204 Critical T Value 2.037 Critical Value for Comparison 0.0417 All 2 means are significantly different from one another.

**LSD All-Pairwise Comparisons Test of trait14 for V**

**V Mean Homogeneous Groups**

A 0.3685 A k 0.3064 B

Alpha 0.05 Standard Error for Comparison 0.0204 Critical T Value 2.037 Critical Value for Comparison 0.0417 All 2 means are significantly different from one another.

**LSD All-Pairwise Comparisons Test of trait14 for P**

**P Mean Homogeneous Groups**

3 0.4474 A

1. 0.3956 A
2. 0.3010 B

0 0.2057 C

Alpha 0.05 Standard Error for Comparison 0.0289

Critical T Value 2.037 Critical Value for Comparison 0.0589 There are 3 groups (A, B, etc.) in which the means are not significantly different from one another.

**LSD All-Pairwise Comparisons Test of trait14 for S*V**

**S V Mean Homogeneous Groups**

control A 0.3942 A Cool A 0.3428 AB control k 0.3269 B Cool k 0.2860 B

Alpha 0.05 Standard Error for Comparison 0.0289

Critical T Value 2.037 Critical Value for Comparison 0.0589 There are 2 groups (A and B) in which the means are not significantly different from one another.

**LSD All-Pairwise Comparisons Test of trait14 for S*P**

**S P Mean Homogeneous Groups**

control 3 0.4737 A Cool 3 0.4211 AB control 1 0.4146 AB Cool 1 0.3766 BC Cool 2 0.3018 CD control 2 0.3003 CD control 0 0.2535 D Cool 0 0.1580 E Alpha 0.05 Standard Error for Comparison 0.0409

Critical T Value 2.037 Critical Value for Comparison 0.0833 There are 5 groups (A, B, etc.) in which the means are not significantly different from one another.

**LSD All-Pairwise Comparisons Test of trait14 for V*P**

**V P Mean Homogeneous Groups**

k 3 0.4633 A A 3 0.4315 AB A 1 0.4069 AB k 1 0.3843 AB A 2 0.3580 BC A 0 0.2775 CD k 2 0.2441 D k 0 0.1340 E

Alpha 0.05 Standard Error for Comparison 0.0409

Critical T Value 2.037 Critical Value for Comparison 0.0833 There are 5 groups (A, B, etc.) in which the means are not significantly different from one another.

**LSD All-Pairwise Comparisons Test of trait14 for S*V*P**

**S V P Mean Homogeneous Groups**

control k 3 0.4860 A control A 3 0.4614 AB Cool k 3 0.4406 ABC control A 1 0.4280 ABC Cool A 3 0.4016 ABC control k 1 0.4012 ABC Cool A 1 0.3858 ABC

Cool k 1 0.3674 BC Cool A 2 0.3596 BCD control A 2 0.3564 BCD control A 0 0.3310 CDE control k 2 0.2442 DEF Cool k 2 0.2440 DEF Cool A 0 0.2240 EF control k 0 0.1760 FG Cool k 0 0.0920 G

Alpha 0.05 Standard Error for Comparison 0.0578

Critical T Value 2.037 Critical Value for Comparison 0.1178 There are 7 groups (A, B, etc.) in which the means are not significantly different from one another.

**LSD All-Pairwise Comparisons Test of Chla for S**

**S Mean Homogeneous Groups** Cool 22.771 A control 20.220 A

Alpha 0.05 Standard Error for Comparison 1.2693 Critical T Value 2.037 Critical Value for Comparison 2.5855 There are no significant pairwise differences among the means.

**LSD All-Pairwise Comparisons Test of Chla for V**

**V Mean Homogeneous Groups**

A 25.163 A

k 17.829 B

Alpha 0.05 Standard Error for Comparison 1.2693 Critical T Value 2.037 Critical Value for Comparison 2.5855 All 2 means are significantly different from one another.

**LSD All-Pairwise Comparisons Test of Chla for P**

**P Mean Homogeneous Groups**

3 23.785 A

1. 22.417 AB
2. 20.822 AB

0 18.960 B

Alpha 0.05 Standard Error for Comparison 1.7950

Critical T Value 2.037 Critical Value for Comparison 3.6564 There are 2 groups (A and B) in which the means are not significantly different from one another. **LSD All-Pairwise Comparisons Test of Chla for S*V**

**S V Mean Homogeneous Groups**

control A 25.322 A Cool A 25.004 A Cool k 20.538 B control k 15.119 C

Alpha 0.05 Standard Error for Comparison 1.7950

Critical T Value 2.037 Critical Value for Comparison 3.6564 There are 3 groups (A, B, etc.) in which the means are not significantly different from one another. **LSD All-Pairwise Comparisons Test of Chla for S*P**

**S P Mean Homogeneous Groups**

Cool 3 25.843 A

Cool 1 23.193 AB Cool 2 22.593 AB control 3 21.726 AB control 1 21.641 AB Cool 0 19.456 B control 2 19.051 B control 0 18.463 B

Alpha 0.05 Standard Error for Comparison 2.5386

Critical T Value 2.037 Critical Value for Comparison 5.1709 There are 2 groups (A and B) in which the means are not significantly different from one another. **LSD All-Pairwise Comparisons Test of Chla for V*P**

**V P Mean Homogeneous Groups**

A 3 27.434 A

A 1 26.142 A

A 2 23.896 AB A 0 23.180 ABC k 3 20.135 BCD k 1 18.692 CDE k 2 17.749 DE k 0 14.739 E

Alpha 0.05 Standard Error for Comparison 2.5386 Critical T Value 2.037 Critical Value for Comparison 5.1709 There are 5 groups (A, B, etc.) in which the means are not significantly different from one another.

**LSD All-Pairwise Comparisons Test of Chla for S*V*P**

**S V P Mean Homogeneous Groups** Cool A 3 28.434 A control A 1 27.386 A control A 3 26.434 A Cool A 1 24.898 A control A 2 24.025 AB Cool A 2 23.766 AB control A 0 23.442 AB Cool k 3 23.252 ABC

Cool A 0 22.918 ABCD

Cool k 1 21.487 ABCD Cool k 2 21.420 ABCD control k 3 17.019 BCDE Cool k 0 15.994 CDE control k 1 15.896 DE control k 2 14.077 E control k 0 13.484 E

Alpha 0.05 Standard Error for Comparison 3.5901

Critical T Value 2.037 Critical Value for Comparison 7.3128 There are 5 groups (A, B, etc.) in which the means are not significantly different from one another.

**LSD All-Pairwise Comparisons Test of Chlb for S**

**S Mean Homogeneous Groups** Cool 4.8677 A control 4.1803 B

Alpha 0.05 Standard Error for Comparison 0.2663 Critical T Value 2.037 Critical Value for Comparison 0.5425 All 2 means are significantly different from one another.

**LSD All-Pairwise Comparisons Test of Chlb for V**

**V Mean Homogeneous Groups**

A 5.0067 A k 4.0414 B

Alpha 0.05 Standard Error for Comparison 0.2663 Critical T Value 2.037 Critical Value for Comparison 0.5425 All 2 means are significantly different from one another.

**LSD All-Pairwise Comparisons Test of Chlb for P**

**P Mean Homogeneous Groups**

3 5.3129 A

1. 4.5906 AB
2. 4.3877 BC

0 3.8050 C

Alpha 0.05 Standard Error for Comparison 0.3766

Critical T Value 2.037 Critical Value for Comparison 0.7672 There are 3 groups (A, B, etc.) in which the means are not significantly different from one another.

**LSD All-Pairwise Comparisons Test of Chlb for S*V**

| **S V Mean Homogeneous Groups** Cool A 5.1151 A control A 4.8982 A Cool k 4.6203 A control k 3.4624 B |  |
| --- | --- |
| Alpha 0.05 Standard Error for Comparison | 0.3766 |
| Critical T Value 2.037 Critical Value for Comparison There are 2 groups (A and B) in which the means are not significantly different from one another. **LSD All-Pairwise Comparisons Test of Chlb for S*P**  **S P Mean Homogeneous Groups**  Cool 3 5.7758 A Cool 1 4.8776 AB control 3 4.8500 AB Cool 2 4.7527 AB control 1 4.3036 BC Cool 0 4.0650 BC control 2 4.0227 BC control 0 3.5450 C | 0.7672 |
| Alpha 0.05 Standard Error for Comparison | 0.5326 |
| Critical T Value 2.037 Critical Value for Comparison There are 3 groups (A, B, etc.) in which the means are not significantly different from one another. **LSD All-Pairwise Comparisons Test of Chlb for V*P**  **V P Mean Homogeneous Groups**  A 3 5.8430 A  A 1 4.9257 AB A 2 4.8321 AB k 3 4.7828 AB A 0 4.4260 B k 1 4.2555 BC k 2 3.9433 BC k 0 3.1840 C | 1.0850 |
| Alpha 0.05 Standard Error for Comparison | 0.5326 |
| Critical T Value 2.037 Critical Value for Comparison There are 3 groups (A, B, etc.) in which the means are not significantly different from one another.  **LSD All-Pairwise Comparisons Test of Chlb for S*V*P**  **S V P Mean Homogeneous Groups**  control A 3 5.9270 A Cool k 3 5.7925 A  Cool A 3 5.7590 A Cool A 0 4.9695 AB control A 1 4.9690 AB Cool A 1 4.8824 AB  Cool k 1 4.8728 AB Cool A 2 4.8497 AB control A 2 4.8144 AB Cool k 2 4.6556 ABC control A 0 3.8824 BC control k 3 3.7731 BC | 1.0850 |

control k 1 3.6381 BC control k 2 3.2310 C control k 0 3.2075 C Cool k 0 3.1605 C

Alpha 0.05 Standard Error for Comparison 0.7533

Critical T Value 2.037 Critical Value for Comparison 1.5344 There are 3 groups (A, B, etc.) in which the means are not significantly different from one another.

**LSD All-Pairwise Comparisons Test of carotenoe for S**

**S Mean Homogeneous Groups**

control 110.56 A Cool 109.07 A

Alpha 0.05 Standard Error for Comparison 7.1503 Critical T Value 2.037 Critical Value for Comparison 14.565 There are no significant pairwise differences among the means.

**LSD All-Pairwise Comparisons Test of carotenoe for V**

**V Mean Homogeneous Groups**

A 160.03 A k 59.60 B

Alpha 0.05 Standard Error for Comparison 7.1503 Critical T Value 2.037 Critical Value for Comparison 14.565 All 2 means are significantly different from one another.

**LSD All-Pairwise Comparisons Test of carotenoe for P**

**P Mean Homogeneous Groups**

3 142.18 A

1. 119.29 B
2. 105.60 B

0 72.19 C

Alpha 0.05 Standard Error for Comparison 10.112

Critical T Value 2.037 Critical Value for Comparison 20.598 There are 3 groups (A, B, etc.) in which the means are not significantly different from one another.

**LSD All-Pairwise Comparisons Test of carotenoe for S*V**

**S V Mean Homogeneous Groups**

control A 162.42 A Cool A 157.64 A Cool k 60.50 B control k 58.70 B

Alpha 0.05 Standard Error for Comparison 10.112

Critical T Value 2.037 Critical Value for Comparison 20.598 There are 2 groups (A and B) in which the means are not significantly different from one another.

**LSD All-Pairwise Comparisons Test of carotenoe for S*P**

**S P Mean Homogeneous Groups**

control 3 143.44 A Cool 3 140.92 AB

Cool 1 126.29 ABC

| control 1 112.28 BCD Cool 2 108.00 CD control 2 103.19 CD control 0 83.31 DE Cool 0 61.06 E |  |
| --- | --- |
| Alpha 0.05 Standard Error for Comparison | 14.301 |
| Critical T Value 2.037 Critical Value for Comparison There are 5 groups (A, B, etc.) in which the means are not significantly different from one another.  **LSD All-Pairwise Comparisons Test of carotenoe for V*P**  **V P Mean Homogeneous Groups**  A 3 192.07 A  A 1 176.65 AB  A 2 160.18 B A 0 111.21 C k 3 92.30 C k 1 61.92 D k 2 51.01 D k 0 33.17 D | 29.129 |
| Alpha 0.05 Standard Error for Comparison | 14.301 |
| Critical T Value 2.037 Critical Value for Comparison There are 4 groups (A, B, etc.) in which the means are not significantly different from one another.  **LSD All-Pairwise Comparisons Test of carotenoe for S*V*P**  **S V P Mean Homogeneous Groups**  control A 3 192.65 A Cool A 3 191.49 A Cool A 1 186.65 A control A 1 166.65 AB Cool A 2 160.67 AB control A 2 159.70 AB control A 0 130.67 BC control k 3 94.23 CD Cool A 0 91.75 CD  Cool k 3 90.36 CD Cool k 1 65.93 DE control k 1 57.92 DE Cool k 2 55.34 DE control k 2 46.68 E control k 0 35.96 E Cool k 0 30.38 E | 29.129 |
| Alpha 0.05 Standard Error for Comparison | 20.224 |
| Critical T Value 2.037 Critical Value for Comparison There are 5 groups (A, B, etc.) in which the means are not significantly different from one another.  **LSD All-Pairwise Comparisons Test of fvm for S**  **S Mean Homogeneous Groups** Cool 0.8020 A control 0.7725 A | 41.195 |
| Alpha 0.05 Standard Error for Comparison | 0.0456 |
| Critical T Value 2.037 Critical Value for Comparison | 0.0929 |

There are no significant pairwise differences among the means.

**LSD All-Pairwise Comparisons Test of fvm for V**

**V Mean Homogeneous Groups** k 0.8106 A A 0.7639 A

Alpha 0.05 Standard Error for Comparison 0.0456 Critical T Value 2.037 Critical Value for Comparison 0.0929 There are no significant pairwise differences among the means.

**LSD All-Pairwise Comparisons Test of fvm for P**

**P Mean Homogeneous Groups**

3 0.8225 A 1 0.7945 A 2 0.7795 A

0 0.7525 A

Alpha 0.05 Standard Error for Comparison 0.0645 Critical T Value 2.037 Critical Value for Comparison 0.1314 There are no significant pairwise differences among the means.

**LSD All-Pairwise Comparisons Test of fvm for S*V**

| **S V** | **Mean** | **Homogeneous Groups** |  |
| --- | --- | --- | --- |
| control k | 0.8512 | A |  |
| Cool A | 0.8340 | A |  |
| Cool k | 0.7700 | AB |  |
| control A | 0.6938 | B |  |
| Alpha |  | 0.05 Standard Error for Comparison | 0.0645 |

Critical T Value 2.037 Critical Value for Comparison 0.1314 There are 2 groups (A and B) in which the means are not significantly different from one another. **LSD All-Pairwise Comparisons Test of fvm for S*P**

| **S P** | **Mean** | **Homogeneous Groups** | |  |
| --- | --- | --- | --- | --- |
| Cool 3 | 0.8320 | A |  |  |
| control 3 | 0.8130 | A |  |  |
| Cool 1 | 0.8090 | A |  |  |
| Cool 2 | 0.7965 | A |  |  |
| control 1 | 0.7800 | A |  |  |
| Cool 0 | 0.7705 | A |  |  |
| control 2 | 0.7625 | A |  |  |
| control 0 | 0.7345 | A |  |  |
| Alpha |  | 0.05 | Standard Error for Comparison | 0.0912 |

Critical T Value 2.037 Critical Value for Comparison 0.1858 There are no significant pairwise differences among the means.

**LSD All-Pairwise Comparisons Test of fvm for V*P**

| **V P** | **Mean** | **Homogeneous Groups** |
| --- | --- | --- |
| k 3 | 0.8445 | A |
| k 1 | 0.8305 | A |
| k 2 | 0.8040 | A |
| A 3 | 0.8005 | A |
| k 0 | 0.7635 | A |
| A 1 | 0.7585 | A |
| A 2 | 0.7550 | A |

A 0 0.7415 A

Alpha 0.05 Standard Error for Comparison 0.0912 Critical T Value 2.037 Critical Value for Comparison 0.1858 There are no significant pairwise differences among the means.

**LSD All-Pairwise Comparisons Test of fvm for S*V*P**

| **S V P** | **Mean** | **Homogeneous Groups** | |  |
| --- | --- | --- | --- | --- |
| control k 3 | 0.8890 | A |  |  |
| control k 1 | 0.8770 | A |  |  |
| Cool A 3 | 0.8640 | A |  |  |
| control k 2 | 0.8430 | A |  |  |
| Cool A 1 | 0.8340 | A |  |  |
| Cool A 2 | 0.8280 | A |  |  |
| Cool A 0 | 0.8100 | A |  |  |
| Cool k 3 | 0.8000 | A |  |  |
| control k 0 | 0.7960 | A |  |  |
| Cool k 1 | 0.7840 | A |  |  |
| Cool k 2 | 0.7650 | A |  |  |
| control A 3 | 0.7370 | A |  |  |
| Cool k 0 | 0.7310 | A |  |  |
| control A 1 | 0.6830 | A |  |  |
| control A 2 | 0.6820 | A |  |  |
| control A 0 | 0.6730 | A |  |  |
| Alpha | 0.05 | | Standard Error for Comparison | 0.1290 |

Critical T Value 2.037 Critical Value for Comparison 0.2628 There are no significant pairwise differences among the means.

**LSD All-Pairwise Comparisons Test of fo for S**

**S Mean Homogeneous Groups**

control 158.04 A Cool 140.04 A

Alpha 0.05 Standard Error for Comparison 9.0125 Critical T Value 2.037 Critical Value for Comparison 18.358 There are no significant pairwise differences among the means.

**LSD All-Pairwise Comparisons Test of fo for V**

**V Mean Homogeneous Groups**

A 150.75 A k 147.33 A

Alpha 0.05 Standard Error for Comparison 9.0125 Critical T Value 2.037 Critical Value for Comparison 18.358 There are no significant pairwise differences among the means.

**LSD All-Pairwise Comparisons Test of fo for P**

**P Mean Homogeneous Groups**

3 193.50 A

1. 173.50 A
2. 146.67 B

0 82.50 C

Alpha 0.05 Standard Error for Comparison 12.746

Critical T Value 2.037 Critical Value for Comparison 25.962 There are 3 groups (A, B, etc.) in which the means are not significantly different from one another.

**LSD All-Pairwise Comparisons Test of fo for S*V**

| **S V Mean Homogeneous Groups**  control A 165.25 A control k 150.83 AB Cool k 143.83 AB  Cool A 136.25 B |  |
| --- | --- |
| Alpha 0.05 Standard Error for Comparison | 12.746 |
| Critical T Value 2.037 Critical Value for Comparison There are 2 groups (A and B) in which the means are not significantly different from one another.  **LSD All-Pairwise Comparisons Test of fo for S*P**  **S P Mean Homogeneous Groups**  control 3 203.17 A Cool 3 183.83 AB control 1 173.67 ABC Cool 1 173.33 ABC control 2 150.00 BC Cool 2 143.33 C control 0 105.33 D Cool 0 59.67 E | 25.962 |
| Alpha 0.05 Standard Error for Comparison | 18.025 |
| Critical T Value 2.037 Critical Value for Comparison There are 5 groups (A, B, etc.) in which the means are not significantly different from one another.  **LSD All-Pairwise Comparisons Test of fo for V*P**  **V P Mean Homogeneous Groups**  k 3 196.33 A A 3 190.67 A k 1 180.33 AB A 1 166.67 AB k 2 148.33 B A 2 145.00 B A 0 100.67 C k 0 64.33 C | 36.716 |
| Alpha 0.05 Standard Error for Comparison | 18.025 |
| Critical T Value 2.037 Critical Value for Comparison There are 3 groups (A, B, etc.) in which the means are not significantly different from one another. **LSD All-Pairwise Comparisons Test of fo for S*V*P**  **S V P Mean Homogeneous Groups**  control A 3 208.67 A control k 3 197.67 AB Cool k 3 195.00 ABC Cool k 1 190.00 ABC control A 1 176.67 ABCD Cool A 3 172.67 ABCD control k 1 170.67 ABCDE Cool A 1 156.67 BCDE control A 2 155.00 BCDE Cool k 2 151.67 BCDE control k 2 145.00 CDE | 36.716 |

Cool A 2 135.00 DEF control A 0 120.67 EFG control k 0 90.00 FGH Cool A 0 80.67 GH

Cool k 0 38.67 H

Alpha 0.05 Standard Error for Comparison 25.491

Critical T Value 2.037 Critical Value for Comparison 51.924 There are 8 groups (A, B, etc.) in which the means are not significantly different from one another.

**LSD All-Pairwise Comparisons Test of fm for S**

**S Mean Homogeneous Groups**

control 712.38 A Cool 647.29 B

Alpha 0.05 Standard Error for Comparison 1.2713 Critical T Value 2.037 Critical Value for Comparison 2.5896 All 2 means are significantly different from one another.

**LSD All-Pairwise Comparisons Test of fm for V**

**V Mean Homogeneous Groups** k 750.46 A A 609.21 B

Alpha 0.05 Standard Error for Comparison 1.2713 Critical T Value 2.037 Critical Value for Comparison 2.5896 All 2 means are significantly different from one another.

**LSD All-Pairwise Comparisons Test of fm for P**

**P Mean Homogeneous Groups**

3 898.00 A

1. 724.67 B
2. 554.67 C

0 542.00 D

Alpha 0.05 Standard Error for Comparison 1.7980 Critical T Value 2.037 Critical Value for Comparison 3.6623 All 4 means are significantly different from one another.

**LSD All-Pairwise Comparisons Test of fm for S*V**

**S V Mean Homogeneous Groups**

control k 876.50 A Cool A 670.17 B Cool k 624.42 C control A 548.25 D

Alpha 0.05 Standard Error for Comparison 1.7980 Critical T Value 2.037 Critical Value for Comparison 3.6623 All 4 means are significantly different from one another.

**LSD All-Pairwise Comparisons Test of fm for S*P**

**S P Mean Homogeneous Groups** Cool 3 961.67 A control 3 834.33 B control 1 799.00 C control 2 658.83 D

Cool 1 650.33 E control 0 557.33 F Cool 0 526.67 G

Cool 2 450.50 H

Alpha 0.05 Standard Error for Comparison 2.5427 Critical T Value 2.037 Critical Value for Comparison 5.1793 All 8 means are significantly different from one another.

**LSD All-Pairwise Comparisons Test of fm for V*P**

**V P Mean Homogeneous Groups**

k 3 972.83 A A 3 823.17 B k 1 818.00 B k 2 771.33 C A 0 644.33 D A 1 631.33 E k 0 439.67 F A 2 338.00 G

Alpha 0.05 Standard Error for Comparison 2.5427

Critical T Value 2.037 Critical Value for Comparison 5.1793 There are 7 groups (A, B, etc.) in which the means are not significantly different from one another. **LSD All-Pairwise Comparisons Test of fm for S*V*P**

**S V P Mean Homogeneous Groups**

control k 3 994.00 A Cool A 3 971.67 B Cool k 3 951.67 C control k 1 932.67 D Cool A 0 910.00 E control k 2 843.33 F control k 0 736.00 G Cool k 1 703.33 H Cool k 2 699.33 H control A 3 674.67 I control A 1 665.33 J Cool A 1 597.33 K control A 2 474.33 L control A 0 378.67 M Cool A 2 201.67 N

Cool k 0 143.33 O

Alpha 0.05 Standard Error for Comparison 3.5959

Critical T Value 2.037 Critical Value for Comparison 7.3246 There are 15 groups (A, B, etc.) in which the means are not significantly different from one another.
